# Targeting CD74 in B-cell non-Hodgkin lymphoma with the antibody-drug conjugate STRO-001

**DOI:** 10.18632/oncotarget.28341

**Published:** 2023-01-12

**Authors:** Xiaofan Li, Cristina Abrahams, Abigail Yu, Millicent Embry, Robert Henningsen, Venita DeAlmeida, Shannon Matheny, Toni Kline, Alice Yam, Ryan Stafford, Trevor Hallam, Mark Lupher, Arturo Molina

**Affiliations:** ^1^Research Development, Sutro Biopharma, South San Francisco, CA 94080, USA; ^2^Clinical Development, Sutro Biopharma, South San Francisco, CA 94080, USA

**Keywords:** CD74, antibody-drug conjugate, non-Hodgkin lymphoma, xenograft models, STRO-001

## Abstract

Overexpression of CD74, a type II transmembrane glycoprotein involved in MHC class II antigen presentation, has been reported in many B-cell non-Hodgkin lymphomas (NHLs) and in multiple myeloma (MM). STRO-001 is a site-specific, predominantly single-species antibody-drug conjugate (ADC) that targets CD74 and has demonstrated efficacy in xenograft models of MM and tolerability in non-human primates. Here we report results of preclinical studies designed to elucidate the potential role of STRO-001 in B-cell NHL. STRO-001 displayed nanomolar and sub-nanomolar cytotoxicity in 88% (15/17) of cancer cell lines tested. STRO-001 showed potent cytotoxicity on proliferating B cells while limited cytotoxicity was observed on naïve human B cells. A linear dose-response relationship was demonstrated *in vivo* for DLBCL models SU-DHL-6 and U2932. Tumor regression was induced at doses less than 5 mg/kg, while maximal activity with complete cures were observed starting at 10 mg/kg. In MCL Mino and Jeko-1 xenografts, STRO-001 starting at 3 mg/kg significantly prolonged survival or induced tumor regression, respectively, leading to tumor eradication in both models. In summary, high CD74 expression levels in tumors, nanomolar cellular potency, and significant anti-tumor in DLBCL and MCL xenograft models support the ongoing clinical study of STRO-001 in patients with B-cell NHL.

## INTRODUCTION

The addition of rituximab to regimens used to treat B-cell non-Hodgkin lymphomas (NHLs) marked a milestone in the treatment of hematologic malignancies [1, 2]. Since that time other novel approaches such as BTK inhibition (eg, ibrutinib) [3], and immunomodulation (eg, lenalidomide) [4], often in combination with chemotherapy, have greatly improved outcomes for most patients with B-cell malignancies. Despite these advances, however, cures remain elusive and effective treatment options are limited or lacking for many patients. The most common type of NHL, diffuse large B-cell lymphoma (DLBCL), is currently associated with a 5-year survival rate of approximately 60% in all patients [5, 6], but only 35% in those having the aggressive ABC-subtype [7]. Median survival in follicular lymphoma (FL), the most common indolent form of NHL [5], has greatly increased, yet it is still considered largely incurable, with a natural history of repeated recurrence and a significant population of poor-prognosis patients subject to relapse within 2 years of initial therapy [8–10]. Among patients with high-risk mantle cell lymphoma (MCL), options are limited and median survival is only approximately 3 years [11–13].

CD74 is a type II transmembrane glycoprotein primarily involved in MHC class II antigen presentation, with additional roles in B-cell maturation, T-cell responses, and macrophage migration inhibitory factor-induced signaling [14]. CD74 expression is limited to HLA class II-positive cells in normal tissues, but its overexpression has been noted in NHL [15, 16] and multiple myeloma (MM) [16–18]. To date, results of only one clinical study of an agent targeting CD74 in B-NHL have been published. A phase I/II trial examining the combination of the anti-CD74 antibody milatuzumab with the anti-CD20 antibody veltuzumab reported an overall response rate of 24% among 34 efficacy-evaluable patients with relapsed/refractory B-NHL [19]. A potentially more efficacious approach to CD74 targeting, namely, the use of an antibody-drug conjugate, could take advantage of the known rapid internalization of surface-expressed CD74 to deliver a potent cytotoxin specifically to targeted cells [20, 21].

We previously reported the cell-free synthesis of STRO-001, a site-specific, predominantly single-species anti-CD74 ADC incorporating a non-cleavable linker-maytansine warhead with a drug-antibody ratio (DAR) of 2 [22]. STRO-001 proved cytotoxic at nanomolar levels in 4 of 6 MM cell lines, and provided significant antitumor activity in the ARP-1 and MM.1S xenograft models of MM. Consistent with the expected pharmacodynamic effect, STRO-001 induced dose-responsive, reversible B-cell and monocyte depletion in cynomolgus monkeys at doses up to 10 mg/kg, with no evidence of off-target toxicity. In order to explore the potential of STRO-001 in NHL, in the present study we investigated CD74 expression in cell types found in bone marrow, evaluated its cytotoxicity in NHL cell lines, and assessed its antitumor efficacy and toxicity in xenograft models of NHL.

## RESULTS

### SP7219 and STRO-001 bind to human and cynomolgus monkey but not mouse CD74

**Figure 1:**
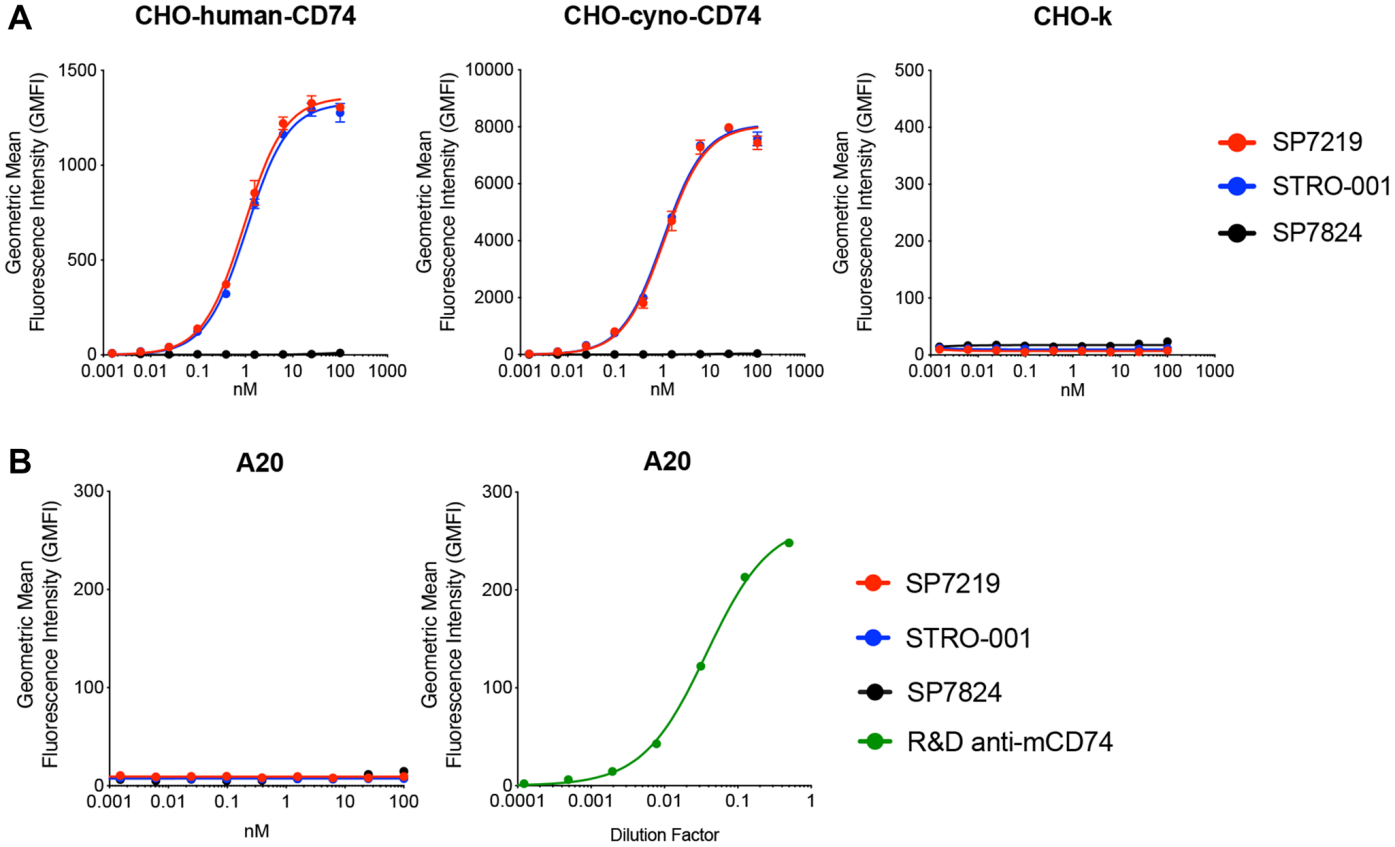
SP7219 and STRO-001 showed similar high affinity binding to human and cynomolgus monkey CD74, no binding to mouse CD74. Anti-GFP antibody SP7824 was used as negative control in the cell binding experiments. (**A**) SP7219 and STRO-001 showed similar high affinity cell binding on CHO cells over-expressing human and cynomolgus monkey CD74. (**B**) SP7219 and STRO-001 showed no binding to mouse B cell line A20. The mouse CD74 expression on A20 was confirmed by a commercial anti-mouse-CD74 antibody from R&D Systems.

In SPR (surface plasmon resonance) based Biacore binding assay, the anti-CD74 antibody SP7219 bound to recombinant human (K_D_ = 1.75 nM) and cynomolgus monkey (K_D_ = 2.70 nM) CD74 ECD (extra-cellular domains), but not to mouse CD74 ECD. SP7219 bound with high affinities to CHO cells transfected with human (K_D_ = 0.93 nM) and cynomolgus monkey (K_D_ = 1.1 nM) CD74. Conjugation to the non-cleavable linker-maytansine warhead did not affect the binding affinity of SP7219 since STRO-001 retained high binding affinity to CHO cells expressing human (K_D_ = 1.1 nM) and monkey CD74 (K_D_ = 1.0 nM) (Figure 1A). Neither SP7219 nor STRO-001 showed any binding to mouse A20 cells expressing mouse CD74 (Figure 1B).

### CD74 expression and STRO-001 cytotoxicity in NHL cell lines

CD74 surface expression was determined in 9 GCB-like DLBCL cell lines, 4 ABC-like DLBCL cell lines, and 4 MCL cell lines. Among the 13 DLBCL cell lines, CD74 copy number varied from high levels (51,000 for GCB-like WSU-DLCL-2; 77,000 for ABC-like OCI-Ly3) to below the lower limit of detection (Table 1). Among the 4 MCL cell lines, CD74 copy numbers varied from 8,000 to 28,000. Consistent with expression in NHL cell lines, CD74 was also observed in 100% (100/100), 100% (28/28), and 94% (73/78) of DLBCL, FL, and MCL human tissue microarray samples, respectively (Supplementary Table 1). CD74 was detected in >70% of cells in 86% (86/100), 79% (22/28), and 63% (49/78) of DLBCL, FL, and MCL samples, respectively. The staining intensity was moderate or marked in 96% (96/100), 96% (27/28), and 63% (49/78) of DLBCL, FL, and MCL samples, respectively (Supplementary Figure 1).

**Table 1:** CD74 cell surface expression level and STRO-001 cytotoxicity in non-hodgkin lymphoma cell lines

Disease	Cell line	SP-7219 Receptor copy number	STRO-001 Cell killing	
			EC50 (nM)	Span (%)
GCB-like DLBCL	WSU-DLCL-2	51,000	0.29	96
	WSU-NHL	50,000	0.80	96
	SU-DHL-4	50,000	0.36	97
	Pfeiffer	47,000	0.54	90
	SU-DHL-6	23,000	0.66	98
	OCI-Ly1	22,000	1.00	93
	HT	21,000	0.34	61
	NUDUL-1	13,000	0.34	98
	OCI-Ly19	<4,000	1.4	54
ABC-like DLBCL	OCI-Ly3	77,000	0.54	95
	U2932	11,000	2.0	79
	RIVA (RI-1)	<4,000	3.7	72
	SU-DHL-2	<4,000	>100	NC
MCL	Mino	28,000	0.68	97
	JVM-2	24,000	1.7	55
	JeKo-1	15,000	0.41	97
	Rec-1	8,000	>100	NC

Abbreviations: ABC: activated B-cell like; DLBCL: diffuse large B-cell lymphoma; GCB: germinal center B-cell like; MCL: mantle cell lymphoma; NC: Not Calculable.

**Figure 2:**
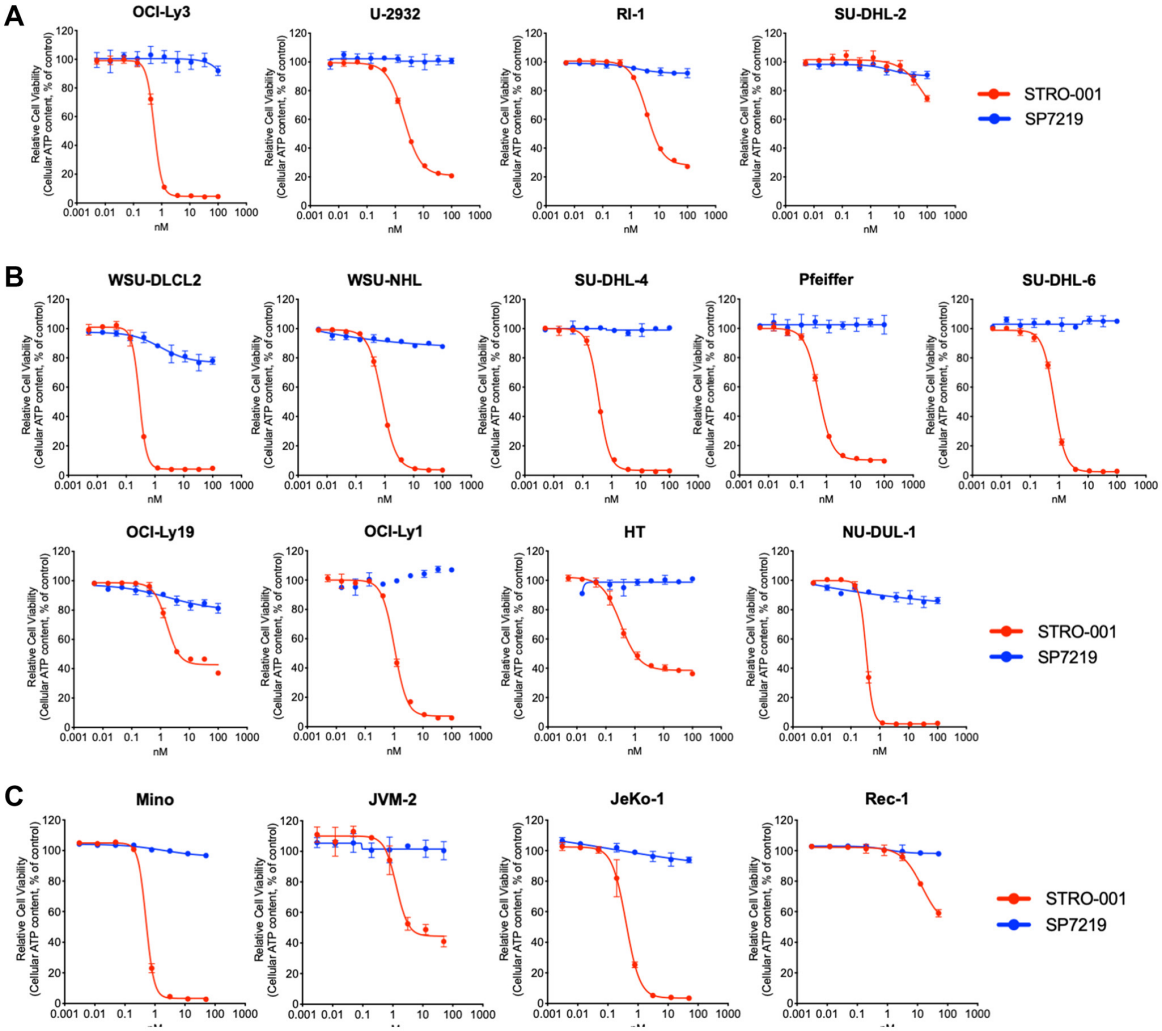
STRO-001 showed potent cell killing activity on NHL cell lines. (**A**) STRO-001 cell killing curves on 4 ABC-like DLBCL cell lines; (**B**) STRO-001 cell killing curves on 9 GCB-like DLBCL cell lines; (**C**) STRO-001 cell killing curves on 4 MCL cell lines.

**Figure 3:**
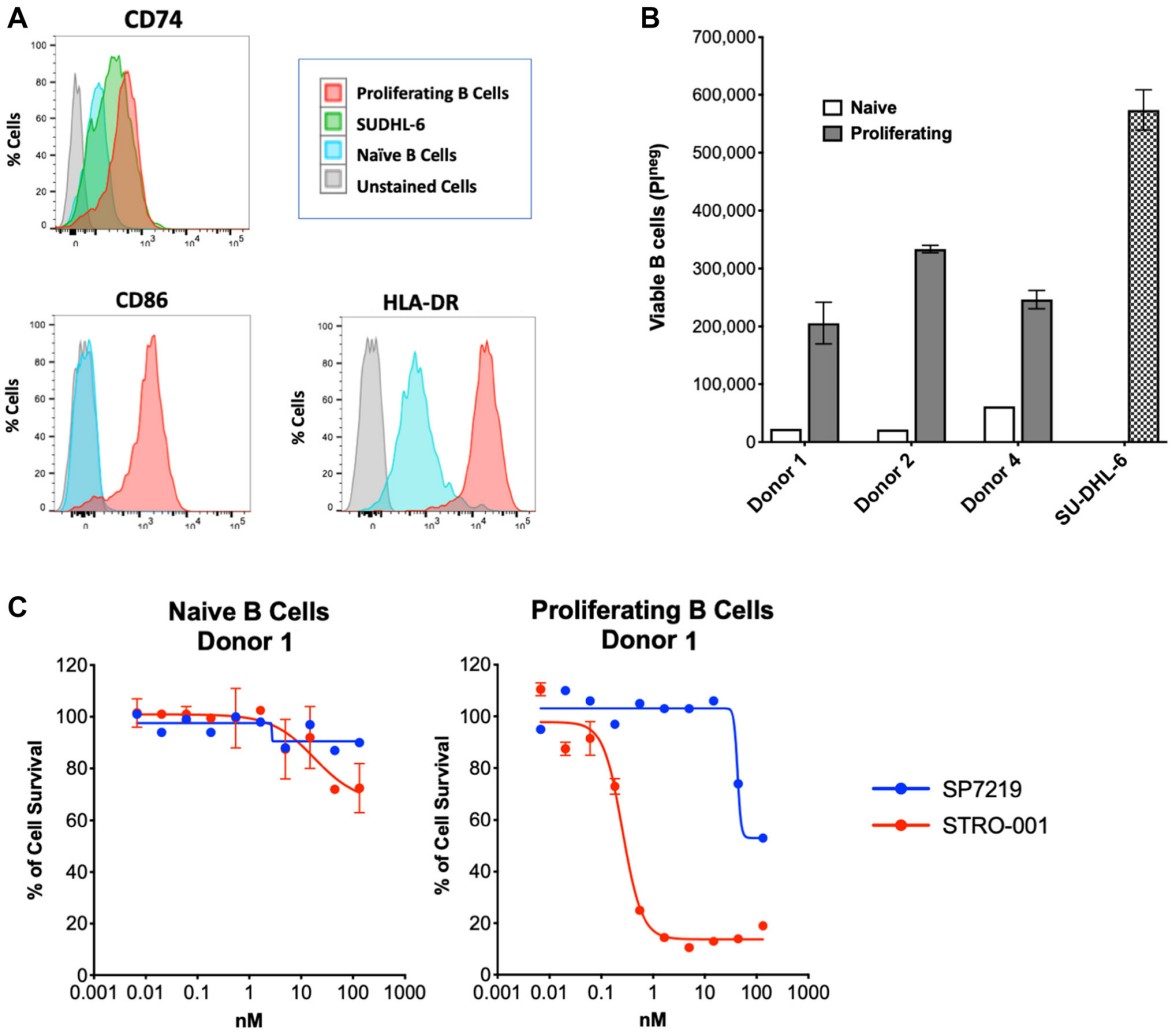
STRO-001 showed potent cell killing activity on proliferating primary human B cells. (**A**) *In vitro* expanded primary B cells showed increased CD86, HLA-DR and CD74 expression compared to naïve B cells. (**B**) *In vitro* expanded B cell showed improved proliferation activity compared to naïve B cells. Cell proliferation was analyzed by flow cytometry using propidium iodide exclusion. (**C**) STRO-001 showed more potent cell killing of proliferating B cells (EC50 = 0.49 nM) than naïve B cells (EC50 = 11 nM).

STRO-001 displayed nanomolar or sub-nanomolar cytotoxicity in 12 of the 13 DLBCL cell lines (Figure 2A and 2B) and 3 of the 4 MCL cell lines (Figure 2C), while the aglycosylated antibody-only portion of STRO-001, SP7219, did not result in cell killing at sub-micromolar levels. A trend (r^2^ = 0.41) toward increased STRO-001 potency with higher CD74 receptor number was observed but appeared to be weak. Notably, nanomolar potency was observed for STRO-001 in 2 of the 3 cell lines with low CD74 expression level below detection limit (OCI-LY19 and RIVA, but not SU-DHL-2, Table 1 and Figure 2A). These results indicate that high CD74 cell surface expression is not required for potent STRO-001 activity, possibly due to the high internalization rate of CD74 leading to efficient warhead delivery [23].

### CD74 expression on different immune cell populations from healthy human and cynomolgus monkey bone marrow aspirates

To determine CD74 expression levels on different immune cell populations from normal bone marrow aspirate (BMA), anti-CD74 antibody SP7219 was conjugated to DBCO-Alexa 647 to generate SP9241, which was used to stain cells isolated from bone marrow aspirates for 5 healthy human and 5 healthy cynomolgus monkey donors. An anti-GFP antibody produced by cell free Xpress+ system was also conjugated to DBCO-Alexa 647 and used as an isotype control (SP9367). The B cells, T cells, NK cells, monocytes, myelocytes, neutrophils, granulocytes and stem cells from human and cynomolgus monkey BMA were labeled with species specific commercial antibodies, respectively (Supplementary Tables 2 and 3). Relative expression of CD74 was expressed as MFI ratio of SP9241 over SP9367 on different immune cell populations. CD74 expression on human B cells was 1.4–2.0-fold higher relative to background, while other cell types did not show CD74 expression above GFP background (Supplementary Table 4). Relative CD74 expression was found to be 2.0–7.3-fold higher on cynomolgus monkey B cells, while other cell types did not show CD74 expression above GFP background (Supplementary Table 5).

### Differential antiproliferative effects of STRO-001 in naïve versus proliferating human B cells

To determine CD74 expression levels and assess STRO-001 cell killing on non-cancerous B cells, naïve B cells from three healthy human donors were isolated and proliferating B cell populations were generated by treating naïve B cells with CellXVivo Human B-cell Expansion Kit (R&D Systems, Minneapolis, MN, USA). Expansion of B cell populations *in vitro* was associated with upregulation of cell surface markers CD86 and HLA-DR, as well as increased CD74 expression, after 5 days of treatment (Figure 3A). B cell expansion led to greater (4–15×) proliferation *in vitro* after 5 days (Figure 3B).

The cytotoxic activity of STRO-001 toward naïve B cells and proliferating B cells was determined using a FACS based cell viability assay after 6 days of treatment. Figure 3C shows cell viability inhibition in naïve B cells (left) and proliferating B cells (right) from the same donor. In naïve B cells, STRO-001 showed minimal cell killing activity with an EC50 value of 18 nM and cell killing span of 34% (Supplementary Table 6). Once B cells were in a proliferative state, STRO-001 induced potent cell killing with an EC50 of 0.26 nM and cell killing span of 87% (Supplementary Table 6). Similar differential cell killing activity was also observed for naïve and proliferating B cells from the other two donors (Supplementary Table 6). Thus STRO-001 preferentially inhibits growth of high CD74-expressing proliferating B cells relative to naïve B cells.

### STRO-001 exhibits robust *in vivo* anti-tumor activity in DLBCL and MCL models

**Figure 4:**
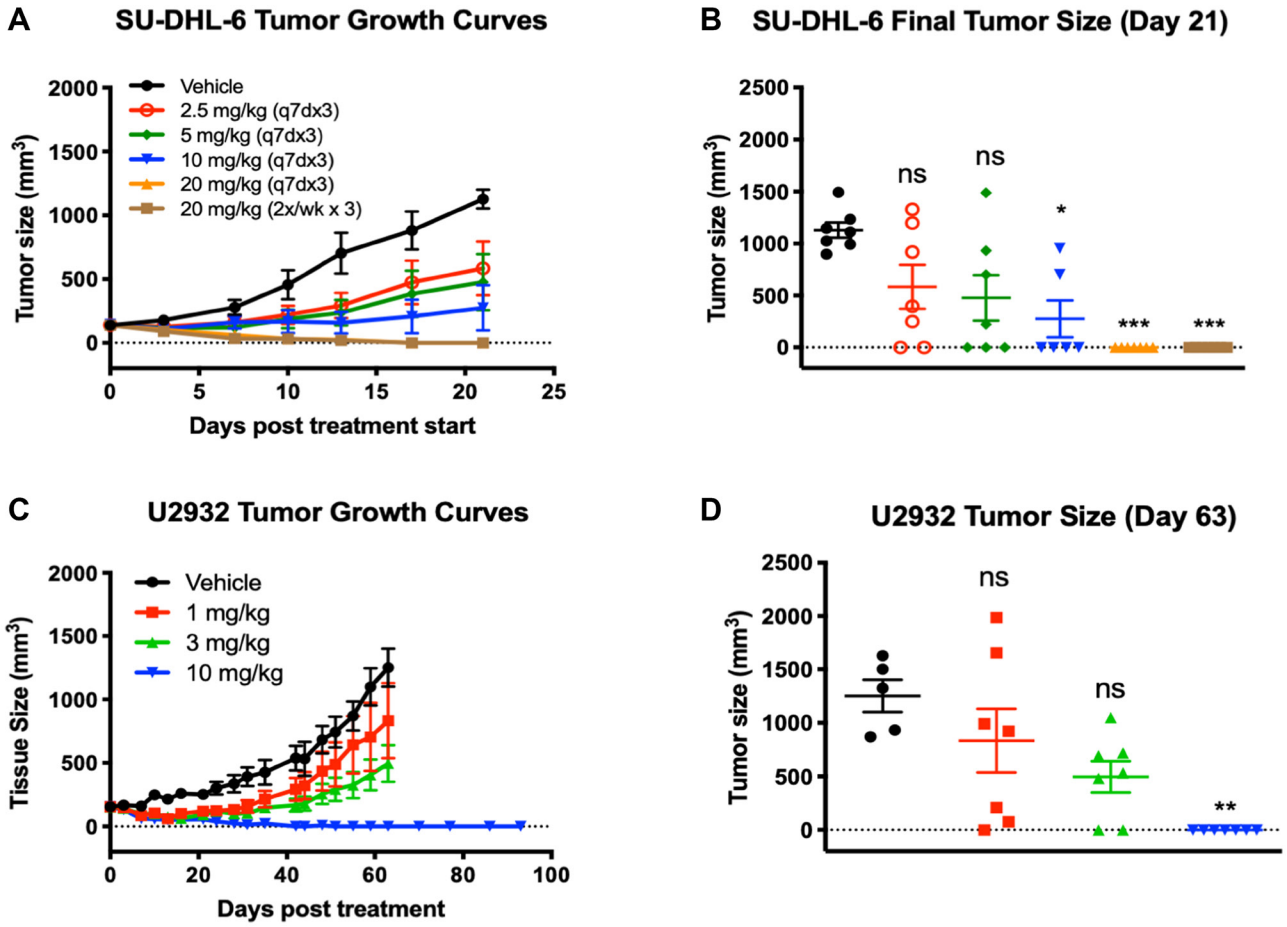
Tumor regression and significant tumor growth inhibition was observed in response to STRO-001 in DBLCL xenograft models. (**A**) SU-DHL-6 tumor growth curves in response to STRO-001 treatment at the indicated dose and dosing interval. (**B**) Scatter plot of individual SU-DHL-6 tumor size on day 21 when mean of control tumors was >1,000 mm^3^. (**C**) U2932 tumor growth curves in response to single-dose STRO-001 treatment at the indicated doses. (**D**) Scatter plot of individual U2932 tumor size on day 63 when mean of control group was >1,200 mm^3^. Both statistical analyses were performed using one-way ANOVA with Kruskal-Wallis and Dunn’s multiple comparisons test.

The *in vivo* anti-tumor activity of STRO-001 was evaluated in NHL models including DLBCL and MCL subcutaneous and disseminated tumor xenografts. Two DLBCL cell lines, SU-DHL-6 and U2932, were chosen based on moderate to low CD74 expression levels (Table 1) and represent the two DLBCL molecular subtypes: germinal center B-cell-like (GBC) and activated B-cell-like (ABC), respectively. Mice bearing established SU-DHL-6 tumors were treated with four different doses of STRO-001 ranging from 2.5 mg/kg to 20 mg/kg administered at weekly intervals for 3 weeks, or at 20 mg/kg twice weekly for 3 weeks. STRO-001 treatment resulted in dose dependent tumor growth inhibition and sustained tumor regression. Moderate anti-tumor activity (55% and 66% tumor growth inhibition [TGI]) characterized by initial tumor regression and tumor stasis for approximately one week was observed at the lower (2.5 mg/kg and 5 mg/kg) doses, respectively (Figure 4A). Although some animals achieved complete responses (e.g., no palpable tumors) in both groups, 2.5 and 5 mg/kg STRO-001 were not statistically significant relative to vehicle control due to the variability in response to treatment (Figure 4B). At 10 mg/kg, STRO-001 treatment approached statistical significance (*p* = 0.053) with 87% TGI and 4 out of 6 complete responders. Maximal activity was observed at 20 mg/kg administered either once-weekly or twice-weekly, led to tumor regression (114% TGI, *p* < 0.001) with all animals (7/7) in both groups remaining tumor free through the end of the study (Figure 4B).

**Figure 5:**
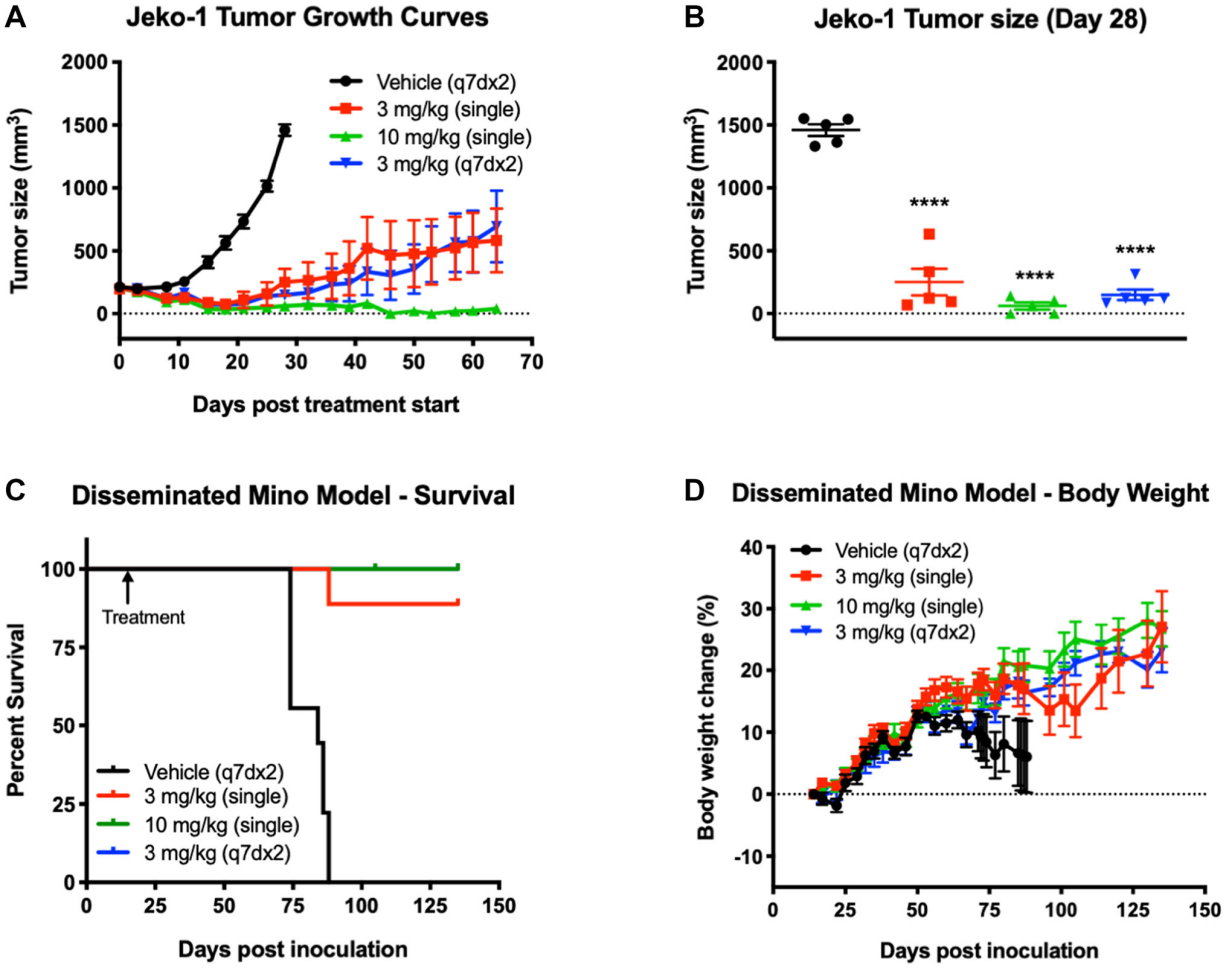
Treatment with STRO-001 eradicates tumor growth and prolongs survival in subcutaneous and disseminated MCL xenograft models. (**A**) Jeko-1 tumor growth curves in response to STRO-001 treatment at the indicated dose and dosing interval. (**B**) Scatter plot of individual Jeko-1 tumor size on day 28 when mean of control group was >1,200 mm^3^. Statistical analysis was performed using one-way ANOVA with Dunnett’s multiple comparisons test. (**C**) Kaplan-Meier curves show the fraction of animals inoculated with Mino cells that survive in response to STRO-001 treatment at the indicated dose and dosing interval. The first day of treatment on Day 11 is indicated by the arrow. Note that the blue 100% survival curve (3 mg/kg, q7dx2) is not visible since it is overlayed by the green 100% survival curve (10 mg/kg, single dose). (**D**) Percent body weight change was calculated relative to animal weight on the first day treatment was administered.

In the U2932 DLBCL ABC model, STRO-001 similarly demonstrated dose dependent TGI with the maximal effective dose resulting in complete tumor regression in all treated animals. Tumor-bearing animals were treated with a single dose of STRO-001 at 1, 3, and 10 mg/kg. Treatment with 1 and 3 mg/kg STRO-001 resulted in 38% and 69% TGI, respectively, but were not statistically significant compared to the vehicle control (Figures 4C and 4D). The highest dose at 10 mg/kg induced complete tumor regression in 7 of 7 mice (114% TGI, *p* < 0.01) (Figures 5A and 5B). In these animals, no tumor regrowth was observed for >90 days post-treatment, indicating potent anti-tumor activity and robust duration of response to a single 10 mg/kg dose of STRO-001 (Figure 4C and 4D).

Two MCL cell lines, Mino and Jeko-1, which express high and low CD74 expression levels (Table 1), respectively, were also assessed for responsiveness to STRO-001. In both MCL models, animals were treated with STRO-001 at 3 mg/kg as a single dose, 3 mg/kg once weekly for 2 weeks (q7dx2), or with a 10 mg/kg single dose. Upon administration of STRO-001 into animals bearing established Jeko-1 subcutaneous tumors, regression was observed in all treatment groups and resulted in 96%, 104%, and 111% TGI in the 3 mg/kg single dose, 3 mg/kg q7dx2, and 10 mg/kg single dose groups, respectively (Figure 5A). On day 28, when the vehicle control group reached approximately 1,500 mm^3^, mean tumor sizes of animals in all three STRO-001 dosing groups were significantly smaller than those in the vehicle control group (*p* < 0.0001) (Figure 5B). Signs of tumor regrowth in both 3 mg/kg STRO-001 dosing groups were evident between days 21 and 25, but at a notably slower rate than for tumors in the vehicle control group (Figure 5A). At 10 mg/kg, 3/5 animals maintained complete tumor regression for up to 64 days post treatment (Figures 5A and 5B).

In the disseminated Mino model, STRO-001 was administered starting on day 14 post-tumor inoculation. The survival endpoint criteria included body weight change loss >20% and clinical signs of morbidity. Mean survival in the vehicle group was 81.3 days, while treatment with STRO-001 at all the doses tested significantly prolonged survival up to 54 days relative to the vehicle control (Figure 5C). Almost all of the STRO-001 treated animals survived (27/28) and remained healthy with increasing body weight until the end of study on Day 135 (Figure 5C and 5D). Cumulatively, treatment with STRO-001 led to a significant reduction in tumor burden, survival benefit and eradication of disease in both the subcutaneous and disseminated MCL models.

## DISCUSSION

In considering novel cancer therapies, the selection of an appropriate target depends on its sufficient expression in or on targeted cells, with limited expression elsewhere. The present study investigated CD74 expression in B-NHL cell lines and patient tumor samples. Biotinylated SP7219, the antibody portion of the ADC STRO-001, which displayed high affinity for human CD74, was used to determine expression levels by immunohistochemistry. We found CD74 expression in 88% (15/17) of DLBCL and MCL cell lines, as well as near-ubiquitous expression in DBCLC, FL and MCL tissue microarrays. These results are consistent with a previous study using a limited number of patient biopsies [15], as well as a recent study reporting CD74 cell surface expression on 404/423 (96%) B-cell NHL biopsy samples [16]. Other investigators have reported that CD74 expression in normal tissues is limited to B cells, monocytes, macrophages, dendritic cells, Langerhans cells, subsets of activated T cells, and thymic epithelium [15, 17, 24–26]. Our study confirmed its expression in B cells from healthy volunteers by FACS and observed that CD74 was upregulated in B cells upon *in vitro* expansion. Taken together, these findings provide a strong rationale for potently and selectively targeting of CD74 in patients with B-NHL.

We previously reported a pharmacodynamics/safety study of STRO-001 in cynomolgus monkey that identified bone marrow toxicity as dose-limiting in this species [22]. At an intravenous dose of 30 mg/kg bone marrow hypocellularity correlating with cytopenia and secondary bacterial infection was noted. To investigate potential on-target toxicity due to CD74 expression in different bone marrow cell types (eg, stem cells), the present study looked at CD74 surface expression in cell types found in cynomolgus monkey as well as human bone marrow. In human bone marrow, CD74 expression was only detected on B cells. In monkey bone marrow, CD74 was detected on B-cells and, to a much lesser extent, NK cells.

STRO-001 displayed nanomolar to sub-nanomolar potency in 88% (15/17) of cell lines, including both GCB-like and ABC-like DLBCL cell lines as well as MCL cell lines. Among these, CD74 surface expression varied considerably. Interestingly, although a weak correlation between CD74 expression and STRO-001 cytotoxicity was noted, the high internalization rate of CD74 may drive robust activity in spite of low target expression as observed in several cell lines. And as expected, the antibody-only portion of STRO-001, SP7219, did not show any cytotoxicity on any of the cell lines tested. Similarly, SP7219 has been shown to lack cytotoxicity in multiple myeloma cell lines [22] and no efficacy in xenograft models of myeloma [27] and B-NHL [28].

In B cells from healthy donors, STRO-001 displayed more potent cytotoxicity in proliferating B cells than in naïve cells. This selectivity was most likely due to higher levels of CD74 surface expression and higher proliferation rate of *in vitro* expanded B cells. Also of note, the major and minor catabolites of STRO-001 containing the maytansinoid warhead, SC246 and SC401, were >15-fold less cytotoxic in MM cell lines, suggesting that the clinical safety profile of STRO-001 may benefit from the consequent minimization of post-catabolic non-specific toxicity (bystander effects) [22].

Consistent with the *in vitro* activity data, STRO-001 demonstrated reproducible and significant therapeutic efficacy in DLBCL and MCL NHL models evaluated *in vivo*. In patients with DLBCL, gene expression [29], disease course, and prognosis [7] are distinct for those with ABC-like and GCB-like disease, with the ABC-like subtype being more aggressive and associated with poorer survival and more limited treatment options. In the SU-DHL-6 xenograft model, STRO-001 displayed dose-dependent tumor growth inhibition and achieved significant efficacy and complete tumor regression in 100% of animals with weekly administration (q7dx3 or 2x/week x3) at the highest dosage of 20 mg/kg. Of note was the lack of toxicity in the SU-DHL-6 tumor bearing animals that received multiple doses of STRO-001 at the 20 mg/kg high doses (data not shown). In contrast, the more aggressive DLBCL-ABC subtype model U2932 and MCL models demonstrated relatively improved sensitivity and responsiveness to STRO-001 treatment. In the U2932 tumors, STRO-001 also exhibited dose-dependent anti-tumor activity, with a single dose of 10 mg/kg resulting in complete tumor regression in 100% of the animals. Meanwhile, significant anti-tumor activity and evidence of cures or tumor eradication was observed with single administration of STRO-001 starting at 3 mg/kg in both the subcutaneous Jeko-1 and disseminated Mino MCL models [30].

Given the established CD74 expression at moderate-to-high levels in the majority of DLBCL, FL, and MCL biopsy samples, the totality of the *in vitro*, *in vivo* and safety data validate the potential clinical effectiveness of the CD74 targeting ADC, STRO-001. A phase 1 study of STRO-001 for the treatment of patients with relapsed/refractory NHL and MM (NCT03424603) has demonstrated preliminary evidence of anti-tumor activity in patients with DLBCL [31].

## MATERIALS AND METHODS

### Cell lines

SU-DHL-6, SU-DHL-2, SU-DHL-4, Pfeiffer, NUDUL-1, HT, JVM-2, Jeko-1, Mino, Rec-1 and A20 cells were purchased from ATCC (American Type Culture Collection, Manassas, VA). OCI-Ly3, U-2932, RIVA, WSU-DLCL2, WSU-NHL, WSU-FSCCL, OCI-Ly1, OCI-Ly19 and OPM2 cells were purchased from The Leibniz Institute DSMZ (German Collection of Microorganisms and Cell Cultures GmbH, Braunschweig, Germany). All the cell lines were maintained in RPMI, high glucose (Corning, Corning, NY, USA) supplemented with 20% heat-inactivated fetal bovine serum (Thermo Scientific, Grand Island, NY, USA), 2 mM GlutaMax (Thermo Scientific, Grand Island, NY, USA), and 1× penicillin/streptomycin (Corning, Corning, NY, USA).

To evaluate the cross-reactivity of STRO-001, CHO-k cells were purchased from ATCC and CHO-humanCD74 and CHO-cynoCD74 stable cell lines were generated by transfecting CHO-k cells with a plasmid containing human or cynomolgus monkey CD74 cDNA sequences. CHO-humanCD74, CHO-cynoCD74 stable cells, CHO-k were maintained in RPMI, high glucose (Corning, Corning, NY, USA) supplemented with 10% heat-inactivated fetal bovine serum (Thermo Scientific, Grand Island, NY, USA), 2 mM glutamax (Thermo Scientific, Grand Island, NY, USA), and 1× Penicillin/streptomycin (Corning, Corning, NY, USA).

### Animals

CB17 SCID mice were obtained from Charles River Laboratories (San Diego, CA, USA). Institutional Animal Care and Use Committee-approved guidelines and protocols were followed in all studies.

### Reagent generation

The procedures used to prepare the ADC STRO-001 and its linker-warhead, SP7219, were described previously [22].

### SP7219 binding to CD74 by surface plasmon resonance (SPR)

The binding affinities of the anti-CD74 antibody SP7219 to the extracellular domain of recombinant mouse, human and cynomolgus monkey CD74 were measured by SPR using a Biacore T200 instrument (GE Life Sciences, Pittsburgh, PA, USA). Test antibodies were injected over a CM4 chip (GE Life Sciences) immobilized with Anti-human Fc polyclonal antibodies (Amine Coupling Kit, GE Life Sciences) at concentrations of 5 μg/mL for 12 seconds at a flow rate of 10 μl/min, followed by a 45 second stabilization period at the same flow rate. The analyte (human CD74-ECD, R&D Systems; cynomolgus CD74-ECD, Lake Pharma custom synthesis; mouse CD74-ECD, R&D Systems) was then allowed to associate to captured antibody for 600 seconds followed by a 600 second dissociation phase at a flow rate of 35 μL/min. The analyte concentrations ranged from 1.6–200 nM (2-fold serially diluted) with 0 nM antigen injections for reference subtraction. The data was fit with the Biacore T200 Evaluation software, using double reference subtraction with a global fit using a 1–1 Langmuir binding model with constant RI of 0. Binding affinity (K_D_, nM) was determined as a ratio of the kinetic rate constants (kd/ka) calculated from the fits of the association and dissociation phases.

### STRO-001 cell binding and antibody binding capacity (ABC) by fluorescence activated cell sorting (FACS)

Cells were harvested and re-suspended in FACS buffer (DPBS buffer supplemented with 1% bovine serum albumin). Cells were incubated on ice with serial dilutions of testing antibodies for 60 minutes. Cells were washed twice with ice-cold FACS buffer and then incubated with 5 μg/ml Alexa 647 labeled donkey anti-human Fc antibody (Jackson ImmunoResearch) on ice for another 60 minutes. For the anti-mouse CD74 antibody conjugated to APC from R&D system (Cat. No. FAB7478A) which has no concentration information available, the antibody was mixed with cells at 1:1 volume ratio and then started 1:4 serial dilution. Unstained cells and cells stained with secondary antibody alone were used as controls. Samples were then washed twice using FACS buffer and analyzed using a BD FACS Canto system. Geometric mean fluorescence intensities (MFI) were fitted using non-linear regression analysis with one site specific binding equation on GraphPad Prism.

CD74 expression level and cell surface antibody binding capacity (ABC) on NHL cell lines was determined using SP7219 conjugated to DBCO-Alexa647 and Quantum™ Simply Cellular^®^ anti-human IgG beads (Bangs Laboratories, Fishers, IN) per the manufacturer’s description as previously described [22].

### ADC cell killing assay in NHL cell lines

Cytotoxicity was measured via a cell proliferation assay in NHL cell lines. Cells were placed in a 384-well flat bottom white polystyrene plate on the assay day. ADCs were formulated at twice the starting concentration in cell culture medium and were filtered through MultiScreen HTS 96-Well Filter Plates (Millipore, Billerica, MA, USA). Filter-sterilized samples were then diluted serially and added to the cells. Plates were cultured for 72 hrs at 37°C in a CO_2_ incubator. To measure cell viability, 30 μL of Cell Titer-Glo^®^ reagent (Promega Corp, Madison, WI) was added to each well, and the plates were processed per product instructions. An ENVISION^®^ plate reader (Perkin-Elmer, Waltham, MA, USA) was used for luminescence measurements. Untreated cells were used as controls to convert luminescence readings to percentage viability. Data fitting employed non-linear regression analysis using a log (inhibitor) versus response, variable slope, 4-parameter fit with Prism (GraphPad Software, Inc., La Jolla, CA, USA). Data were expressed as percent relative cell viability versus ADC dose in nM.

### Immunohistochemistry (IHC) staining of NHL patient samples

The study was conducted at Nova Pathology (Bellingham, WA) in accordance with internal SOPs. Commercially sourced, paraffin-embedded human tissue microarrays (TMAs) representing DLBCL, FL, and MCL samples were analyzed and scored by Michael J Tomlinson, DVM, Ph.D. IHC analysis was performed using biotinylated anti-CD74 antibody (Bio-SP7219) [22]. Frequency of labeling was categorized as negative (no labeled cells), rare (0–30% labeled), occasional (31–70% labeled) and frequent (>70% labeled). Intensity of labeling was categorized as negative (no labeled cells), 1 (mild staining), 2 (moderate staining), and 3 (marked staining).

### CD74 expression on different immune cell populations from human and cynomolgus monkey bone marrow aspirates (BMA)

Whole BMA (3 mL each) from 5 healthy human donors not treated with GCSF were obtained through AllCells, LLC (Alameda, CA, USA). Whole BMA (2 mL each) from 5 healthy cynomolgus monkeys were obtained through Worldwide Primates, Inc. (Miami, Florida). Samples were assayed on the day received. Briefly, samples were diluted 1:4 with DPBS, washed with 20 mL DPBS, then resuspended in 10 ml ACK lysing buffer (Lonza Bioscience, Basel, Switzerland) and incubated at room temperature for 10 minutes to remove red blood cells. Cells were then washed with 25 mL DPBS, re-suspended in FACS buffer and blocked with 5 uL of human FC block buffer (Becton Dickinson, San Jose, CA, USA) for 15 minutes on ice. The various cell types from human or cynomolgus monkey BMA were labeled with commercial antibodies to human (Supplementary Table 5) or cynomolgus monkey (Supplementary Table 6). The cell types were also co-stained with 50 nM fluorescent labeled anti-CD74 antibody (SP9241) for CD74 expression or 50 nM fluorescent labeled anti-GFP antibody (SP9367) for background control. Cells were then washed twice with FACS buffer and re-suspended in FACS buffer with propidium iodide. FACS analysis was then carried out as described above.

### STRO-001 cytotoxicity on naïve and activated B cells from healthy donors

To assess the effects of STRO-001 on human B cells, peripheral blood mononuclear cells (PBMC) from three different healthy human donors (Buffy Coats from Stanford Blood Center, Palo Alto, CA, USA) were isolated by density gradient centrifugation (Nycoprep 1.077, Cosmo Bio USA, Carlsbad, CA, USA). Untouched human B cells were purified by negative isolation (B Cell Isolation Kit II, Miltenyi Biotec, San Diego, CA, USA). B cells were cultured at 0.1 × 10^6^ cells per well in 96-well plates with or without *in vitro* expansion reagents (CellXVivo Human B-cell Expansion Kit, R&D Systems) in RPMI media containing 10% heat inactivated fetal bovine serum (SH30070.03HI, HyClone, GE Healthcare Life Sciences). Next day, serial dilution of STRO-001 and control unconjugated Ab were added to naïve and proliferating B cells and cultured for 6 days. Cell killing was assessed by flow cytometry using propidium iodide (PI) staining to identify dead B cells and absolute counting beads (flow cytometry absolute count standard, Bangs Laboratories, Fishers, IN, USA) to quantify viable B-cell numbers. In addition, surface expression of CD80, CD86, HLA-DR and CD74 on B-cell was confirmed after 4 to 5 days by antibodies from eBioscience.

### NHL xenograft models

In the SU-DHL-6, U2932, and Jeko-1 models, a 1:1 mixture of matrigel and 1 × 10^7^ tumor cells were implanted subcutaneously into the right flank of isoflurane-anesthetized female CB17 SCID mice. Randomization (*n* = 5–7 per group) and start of treatment began when the average tumor size was 150 mm^3^ for SU-DHL-6 and U2932, and 200 mm^3^ for Jeko-1 (designated as Day 0 post treatment). Body weight and tumor size were monitored 1–2 times per week until tumors were >1,000 mm^3^ for SU-DHL-6 and >1,200 mm^3^ for U2932 and Jeko-1.

For the Mino model, female CB17 SCID mice were inoculated with 1 × 10^7^ Mino cells via tail vein injection. Randomization (*n* = 9–10 per group) and start of treatment were initiated 14 days post inoculation. Survival endpoint was characterized by >20% body weight loss and clinical signs including lethargy or morbidity. Animals were monitored at least twice a week for body weight changes and onset of MM clinical signs including changes in posture, fur, gait, and mobility resulting from hind limb paralysis. Kaplan–Meier curves depict animal survival characterized by substantial body weight change or moribundity.

In all models, STRO-001 and vehicle control (PBS) were administered intravenously (IV) at the intervals and doses indicated. Body weight change (BWC) was calculated relative to weight on the first day of treatment using the formula BWC = ([W_current_ − W_initial_]/W_initial_) × 100 where W_intial_ is the weight on the first day of treatment (day 0 post-treatment). Significant toxicity was defined as a >20% decrease in animal weight, at which point affected animals were euthanized. All graphs are presented as mean or individual values ± standard error of measurement (SEM). All results were reproduced in at least 2 independent studies, and representative data from one experiment is shown.

### Statistical analysis

Statistical analyses of final tumor sizes were performed using one-way analysis of variance (ANOVA) with Dunnett’s multiple comparison test using Prism software (version 6). When Brown-Forsythe and Bartlett’s tests showed significant data heterogeneity, non-parametric analysis was subsequently performed using Kruskal-Wallis test with Dunn’s multiple comparisons test. A probability of <5% (*p* < 0.05) was considered statistically significant. Annotations for *p*-values are: ^*^ *p* < 0.05; ^**^ *p* < 0.01; ^***^ *p* < 0.001, ^****^ *p* < 0.0001; ns: not significant.

## SUPPLEMENTARY MATERIALS



## Abbreviations

ABC: activated B cell
ADC: antibody-drug conjugate
BMA: bone marrow aspirate
CHO: Chinese hamster ovary
DAR: drug-antibody ratio
DLBCL: diffuse large cell lymphoma
DMSO: dimethyl sulfoxide
DPBS: Dulbecco’s phosphate buffered saline
FACS: fluorescence activated cell sorting
FL: follicular lymphoma
GCB: germinal center B cell
mAb: monoclonal antibody
MCL: mantle cell lymphoma
MM: multiple myeloma
NHL: non-Hodgkin lymphoma
NC: not calculable
NS: not significant
SCID: severe combined immunodeficiency disease
TGI: tumor growth inhibition
