# Supplementary material for: Targeting CD74 in B-cell non-Hodgkin lymphoma with the antibody-drug conjugate STRO-001
Source: Oncotarget. 2023 Jan 12;14:1–13. doi: 10.18632/oncotarget.28341 (PMC9836384; doi:10.18632/oncotarget.28341)
Supplement: Supplementary file 1 [file oncotarget-14-28341-s001.pdf]

Targeting CD74 in B-cell non-Hodgkin lymphoma with the antibody-drug conjugate STRO-001

SUPPLEMENTARY MATERIALS

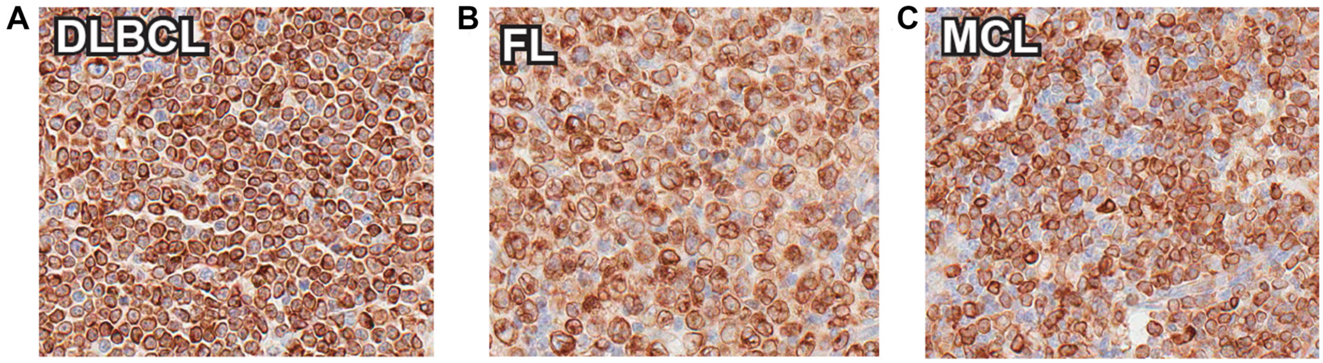

Supplementary Figure 1: CD74 expression in NHL tissue microarrays. Representative IHC images at 40× magnification of CD74 staining in DLBCL (**A**), FL (**B**) and MCL (**C**) tissue microarrays.

Supplementary Table 1: CD74 is highly expressed in NHL tissue microarrays by immunohistochemistry

Histology	Frequency				Intensity			
	0	1	2	3	0	1	2	3
DLBCL	0/100	4/100	10/100	86/100	0/100	4/100	31/100	65/100
FL	0/28	1/28	5/28	22/28	0/28	1/28	10/28	17/28
MCL	5/78	12/78	12/78	49/78	5/78	24/78	32/78	17/78

Specific binding of the biotinylated anti-CD74 antibody to the tumor microarrays was analyzed and scored. Staining frequency: 0: no labeled cells; 1: rare (0–30% of cells); 2: occasional (31–70% of cells); 3: frequent (>70% of cells). Staining intensity: 0: no labeled cells; 1: mild; 2: moderate; 3: marked. Abbreviations: DLBCL: diffuse large B-cell lymphoma; FL: follicular lymphoma; MCL: mantle cell lymphoma.

Supplementary Table 2: Commercial antibodies used to label different immune cell populations for human bone marrow aspirate

Set	Cell type	Immunotype	Antigen	Fluor	Reactivity	Vendor	Clone
1	T cells	CD3+	CD3	PE	Hu, Cy	BD Biosciences	SP34
	B cells	CD20+	CD20	FITC	Hu, Cy	BD Biosciences	L27
2	Granulocytes	CD66b+	CD66b	FITC	Hu	eBio	G10F5
3	NK cells	CD56+	CD56	PE	Hu, Cy	eBIo	TULY56
	Monocytes	CD14+	CD14	PE	Hu, Cy	BD Biosciences	M5E2
	Neutrophils	CD16+	CD16	FITC	Hu	Biolegend	3G8
4	Myelocyte	CD33+/CD16−	CD16	PE	Hu	Biolegend	3G8
			CD33	FITC	Hu, Cy	Miltenyi	REA775
5	Stem cells	CD34+	CD34	FITC	Hu, Cy	Biolegend	561

Abbreviations: Cy: cynomolgus monkey; FITC: fluorescein isothiocyanate; Hu: human; PE: phycoerythrin.

Supplementary Table 3: Commercial antibodies used to label different immune cell populations for cynomolgus monkey bone marrow aspirate

Set	Cell type	Immunotype	Antigen	Fluor	Reactivity	Vendor	Clone
1	T cells	CD3+	CD3	PE	Hu, Cy	BD Biosciences	SP34
	B cells	CD20+	CD20	FITC	Hu, Cy	BD Biosciences	L27
2	NK cells	CD56+	CD56	PE	Hu, Cy	eBio	TULY56
3	Monocytes	CD14+	CD14	PE	Hu, Cy	BD Biosciences	M5E2
4	Myelocytes	CD33+	CD33	FITC	Hu, Cy	Miltenyi	REA775
5	Stem cells	CD34+	CD34	FITC	Hu, Cy	Biolegend	561

Abbreviations: Cy: cynomolgus monkey; FITC: fluorescein isothiocyanate; Hu: human; PE: phycoerythrin.

Supplementary Table 4: Relative CD74 expression on different immune cell populations in bone marrow from five healthy human donors

Cell type	Donor 1	Donor 2	Donor 3	Donor 4	Donor 5
B cells					
Percentage	1.3	0.9	1.0	0.9	1.2
MFI ratio	1.8	2.0	1.4	1.7	1.6
T cells					
Percentage	4.9	6.3	6.3	10.1	6.4
MFI ratio	0.4	0.6	0.5	0.6	0.5
Granulocytes					
Percentage	81.5	67.0	75.8	67.4	65.0
MFI ratio	0.7	0.7	0.8	0.8	0.7
NK cells					
Percentage	1.5	2.0	1.3	3.5	2.5
MFI ratio	0.5	0.6	0.5	0.6	0.7
Monocytes					
Percentage	2.6	4.6	3.7	5.6	5.5
MFI ratio	0.5	1.0	0.6	1.0	0.9
Neutrophils					
Percentage	17.8	47.0	36.4	37.4	37.2
MFI ratio	0.6	0.7	0.7	0.7	0.7
Myelocytes					
Percentage	10.4	8.5	11.2	16.5	11.4
MFI ratio	0.9	0.9	0.9	0.9	0.9
Stem cells					
Percentage	0.8	0.8	0.9	1.0	1.0
MFI ratio	0.5	0.5	0.4	0.6	0.4

Relative CD74 expression was indicated by MFI ratio of fluorescent labeled ant-CD74 antibody (SP9241)/fluorescent labeled ant-GFP antibody (SP9367).

Supplementary Table 5: Relative CD74 expression on different immune cell populations in bone marrow from five healthy cynomolgus monkeys

Cell type	Donor 1	Donor 2	Donor 3	Donor 4	Donor 5
B cells					
Percentage	5.6	3.2	11.8	5.3	5.3
MFI ratio	5.2	2.0	7.3	5.2	4.3
T cells					
Percentage	21.5	8.2	23.3	8.0	8.0
MFI ratio	1.1	0.9	1.0	1.0	0.9
NK cells					
Percentage	9.5	9.0	4.2	8.1	12.4
MFI ratio	1.7	1.3	2.1	1.6	1.3
Monocytes					
Percentage	22.1	17.0	16.5	24.4	24.0
MFI ratio	1.1	1.1	1.2	1.1	1.1
Myelocytes					
Percentage	4.0	4.7	3.7	7.1	5.1
MFI ratio	1.1	1.1	1.0	1.0	1.0
Stem cells					
Percentage	0.1	0.4	0.2	0.3	0.8
MFI ratio	NE*	NE*	NE*	NE*	1.6

Relative CD74 expression was indicated by MFI ratio of fluorescent labeled ant-CD74 antibody (SP9241)/fluorescent labeled ant-GFP antibody (SP9367). *Not evaluable; insufficient number of cells analyzed to determine MFI ratio.

Supplementary Table 6: Antiproliferative activity of STRO-001 in naïve and proliferating primary human B cells

Cell type	Donor #1		Donor #2		Donor #4	
	EC50 (nM)	Span (%)	EC50 (nM)	Span (%)	EC50 (nM)	Span (%)
Naive B cells	18	34	1.9	41	10	31
Proliferating B cells	0.26	87	0.23	97	0.49	92
